# Ethnobotany, Phytochemistry, Biological Activities, and Health-Promoting Effects of the Genus *Bulbophyllum*

**DOI:** 10.1155/2022/6727609

**Published:** 2022-03-07

**Authors:** Javad Sharifi-Rad, Cristina Quispe, Abdelhakim Bouyahya, Naoual El Menyiy, Nasreddine El Omari, Md Shahinozzaman, Mim Ara Haque Ovey, Niranjan Koirala, Mamata Panthi, Andrea Ertani, Silvana Nicola, Natallia Lapava, Jesús Herrera-Bravo, Luis A. Salazar, Sushil Changan, Manoj Kumar, Daniela Calina

**Affiliations:** ^1^Facultad de Medicina, Universidad del Azuay, Cuenca, Ecuador; ^2^Facultad de Ciencias de la Salud, Universidad Arturo Prat, Avda. Arturo Prat 2120, Iquique 1110939, Chile; ^3^Laboratory of Human Pathologies Biology, Department of Biology, Faculty of Sciences, and Genomic Center of Human Pathologies, Faculty of Medicine and Pharmacy, Mohammed V University in Rabat, Rabat, Morocco; ^4^Laboratory of Pharmacology, National Agency of Medicinal and Aromatic Plants, Taounate 34025, Morocco; ^5^Laboratory of Histology, Embryology, and Cytogenetic, Faculty of Medicine and Pharmacy, Mohammed V University in Rabat, Rabat, Morocco; ^6^Department of Nutrition and Food Sciences, University of Maryland, College Park, MD 20742, USA; ^7^Department of Biochemistry and Molecular Biology, University of Dhaka, Dhaka-1000, Bangladesh; ^8^Department of Natural Products Research, Dr. Koirala Research Institute for Biotechnology and Biodiversity, Kathmandu 44600, Nepal; ^9^Laboratory of Biotechnology, Faculty of Science and Technology, University of Macau, Macau SAR 999078, China; ^10^Department of Agricultural, Forest and Food Sciences, University of Turin, Turin, Italy; ^11^Medicine Standardization Department of Vitebsk State Medical University, Vitebsk, Belarus; ^12^Departamento de Ciencias Básicas, Facultad de Ciencias, Universidad Santo Tomas, Santiago, Chile; ^13^Center of Molecular Biology and Pharmacogenetics, Scientific and Technological Bioresource Nucleus, Universidad de La Frontera, Temuco 4811230, Chile; ^14^Division of Crop Physiology, Biochemistry and Post-Harvest Technology, ICAR-Central Potato Research Institute, Shimla, Himachal Pradesh, India; ^15^Chemical and Biochemical Processing Division, ICAR-Central Institute for Research on Cotton Technology, Mumbai 400019, India; ^16^Department of Clinical Pharmacy, University of Medicine and Pharmacy of Craiova, 200349 Craiova, Romania

## Abstract

The genus *Bulbophyllum* is of scientific interest due to the phytochemical components and diverse biological activities found across species of the genus. Most *Bulbophyllum* species are epiphytic and located in habitats that range from subtropical dry forests to wet montane cloud forests. In many cultures, the genus *Bulbophyllum* has a religious, protective, ornamenting, cosmetic, and medicinal role. Detailed investigations into the molecular pharmacological mechanisms and numerous biological effects of *Bulbophyllum* spp. remain ambiguous. The review focuses on an in-depth discussion of studies containing data on phytochemistry and preclinical pharmacology. Thus, the purpose of this review was to summarize the therapeutic potential of *Bulbophyllum* spp. biocompounds. Data were collected from several scientific databases such as PubMed and ScienceDirect, other professional websites, and traditional medicine books to obtain the necessary information. Evidence from pharmacological studies has shown that various phytoconstituents in some *Bulbophyllum* species have different biological health-promoting activities such as antimicrobial, antifungal, antioxidant, anti-inflammatory, anticancer, and neuroprotective. No toxicological effects have been reported to date. Future clinical trials are needed for the clinical confirmation of biological activities proven in preclinical studies. Although orchid species are cultivated for ornamental purposes and have a wide traditional use, the novelty of this review is a summary of biological actions from preclinical studies, thus supporting ethnopharmacological data.

## 1. Introduction

Orchids are the largest group of angiosperms consisting of nearly 28,000 species with over 736 genera [1]. Although orchids are found in natural habitats in several parts of the world, their presence is decreasing due to great demand by the population [2]. Due to habitat destruction and indiscriminate collection, Orchid species are at a steady loss [3]. One of the most represented genera is *Bulbophyllum* Thouars (*Orchidaceae*: subfamily *Epidendroideae*, subtribe *Bulbophyllinae*) with ca. 2,200 species distributed in Africa and Asia (China, Nepal, India, Thailand, Laos, and Vietnam) [4]. The taxonomic history of *Bulbophyllum* has been complex since its establishment [5]. The great dimension and indefinite infrageneric systematics of this genus bring significant difficulties, reducing evolution, ecology, and morphology research [6]. Taxonomists have reported at least 24 closely related genera, classified based on floral morphology [7]. Only recently, molecular biology techniques have allowed recognizing this genus as monophyletic, i.e., belonging to a common ancestor [8].

Most *Bulbophyllum* species are epiphytic and located in habitats that range from subtropical dry forests to wet montane cloud forests [9, 10]. They exclusively obtain water and nutrients from air, rain, and debris and thus must be able to overcome difficult environmental conditions by storing water in the pseudobulbs [11]. In addition to the economic importance attributable to ornamental uses, herbal medicinal properties of phytochemical substances of biological interest (such as flavonoids, sterols-terpenoids, and phenolic acids) in *Bulbophyllum* species have also been reported [12, 13]. Several studies report both the phytochemical compounds and the molecules' biological effects extracted from *Bulbophyllum* leaf, pseudobulb, and root, for traditional medicine treatments [14].

In a continuous effort by researchers to discover the biological activities of Bulbophyllum species, we describe and summarize recent research in ethnobotanical knowledge, phytochemistry, and pharmacological properties, along with the limitations of the research in our article.

## 2. Methodology

To conduct this review, data were collected from research in several scientific databases, such as PubMed and ScienceDirect, using the following MeSH terms: “Orchidaceae/chemistry∗,” “Plant Extracts/chemistry,” “Drugs, Herbal/chemistry,” “Molecular Structure,” “Anti-Inflammatory Agents/chemistry,” “Antineoplastic Agents/chemistry.” The study included documents written in English language that discussed ethnopharmacology, phytochemistry, and biological activities of these species. The taxonomy of the species has been validated according to the Plant List [15, 16].

## 3. Botany, Species, and Distribution

### 3.1. Botanical Aspects

Plants of the genus *Bulbophyllum* are epilithic herbs, sympodial with roots creeping over the surface of the substrate or aerial, and filamentous to fibrous [17]. Orchid plants, such as Australian *Bulbophyllum minutissimum*, differ in size and weight from a gram to a few millimetres (1–1.5 mm across) [18]. The stems differentiate into rhizome and pseudobulb, and the leaves may be ovate, lanceolate, or orbiculate and variable in size on an individual plant [19]. Leaves are terminal on the pseudobulb (one or several per shoots) and conduplicate to the substrate. Recently, Piazza et al. [20] studied the vegetative anatomy of 13 species of *Bulbophyllum* belonging to sections *Didactyle* and *Xiphizusa* to elucidate the anatomical characters between and among the sections. The results revealed the anatomical differences among the species of both lipophilic secretion sections in young leaf trichomes and the presence of xeromorphic characters. Adaptations of both species to different environmental conditions were among the main differences described by the authors [20].

The inflorescence is racemose and presents many lateral flowers emerging from the rhizome, often at the base of the pseudobulb [19]. Some orchid species in the genus *Bulbophyllum* show an elaborated floral architecture, in addition to their characteristic floral odours that attract and bring specific pollinators [21]. The dorsal sepal is free, similar, or smaller than the lateral sepals. The latter are united basally to each other and a *column* foot forms a *mentum*, free or fused further [19]. The lateral petals are free and smaller than the dorsal sepals, and the lip of the flowers is trilobed with a large callus. In some species, the lip flaps with the wind, to simulate a fly shaking its wings to attract insects [9]. The *labellum* is hinged to the tip of the column foot; the lamina is either not lobed, obscurely 3-lobed, unornamented, or with 2 longitudinal keels. The column lacks the free filament and style; the column wings are fused to the column and reduced to teeth that project beside the anther [9]. The ovary, composed of three fused carpels and the mature seed pod, opens down the middle between the lines of juncture. The ovules are arranged along the ridges inside the ovary and do not develop until sometime after the flower [9]. Orchid flowers attract specific insect species by deceiving males with an imitation signal of female odour and mimetic appearance [22]. For example, the ginger orchid (*Bulbophyllum patens* King) flower releases a ginger essence called zingerone that attracts fruit flies sensitive to methyl eugenol and raspberry ketone [22, 23]. Also, the flowers of *Bulbophyllum apertum* Schltr. release the volatile compound raspberry ketone with the function of attracting raspberry ketone-sensitive *Bactrocera* species [23].

The seeds of *Bulbophyllum* species consist of a dry, outer coat enclosing a small mass of undifferentiated cells that form a proembryo [17]. They are fusiform, spindle, and narrowly ellipsoidal shaped and less than 1 mm in length without endosperm [24]. This extremely small and light unit can easily be carried in through air currents and may travel long distances before coming to rest [17]. Seed volume is related to seed size and *Bulbophyllum* species, and the higher seed volume is a result of greater width rather than *testa* length [24]. In natural conditions, the seeds have specific germination requirements provided by mycorrhizal fungi. For instance, seeds infected by a specific fungus can either germinate or be destroyed [25]. There are different seed morphologies among the plants of the genus *Bulbophyllum*. For example, *Bulbophyllum mysorense* seeds are transparent, short, and spindle to oblong with blunt ends, and the *testa* cells have marginal clavate ridges, smooth on the outer face with longitudinally oriented cells resembling a twisted rope [26].

### 3.2. *Bulbophyllum* Species and Cultivation

Every year, some new *Bulbophyllum* species are described, making this genus grow steadily [27]. Due to the extraordinary diversity, a general description would be too extensive [1, 27]. Over 50 generic names have been proposed, in addition to *Bulbophyllum* [19].

In 2005, the Federal Ministry of Agriculture, Forestry, Environment, and Water Management as the CITES Management Authority of Austria elaborated three lists (names, accepted names, and the checklist) for the *Bulbophyllum* genus, produced by the Botanical Garden of Vienna [28]. The list is an approach to handle the vast number of species of this genus [28]. Recently, *Bulbophyllum* is into a single genus [7, 19]. In 2019, the World Checklist of Selected Plant Families [29] published an updated and exhaustive list that comprises over 2000 epiphytic *Bulbophyllum* species. There have been many efforts to split off segregate genera based on morphological characteristics. As reported by the American Orchid Society [30], some of the common *Bulbophyllum* species are as follows: *Bulbophyllum dearei, Bulbophyllum echinolabium, Bulbophyllum falcatum, Bulbophyllum fascinator, Bulbophyllum flabellum-veneris, Bulbophyllum guttulatum, Bulbophyllum lasiochilum, Bulbophyllum lobbii, Bulbophyllum longissimum, Bulbophyllum makoyanum, Bulbophyllum medusae,* and *Bulbophyllum putidum*. Two hybrids, Bulbophyllum Daisy Chain and Bulbophyllum Elizabeth discovered recently, are particularly attractive [30]. Among this ecological group, new taxa were also discovered: *Bulbophyllum cariniflorum* Reichenbach var. *orlovii* Aver., *Bulbophyllum sonii* Aver. and N. V. Duy, *Bulbophyllum ustulata* Aver., *Bulbophyllum cariniflorum*, *Bulbophyllum flavescens* Lindley, *Bulbophyllum ovatum* Seidenfaden, *Bulbophyllum physocoryphum* Seidenfaden, and *Bulbophyllum wendlandianum* Dammer.

Regarding the environmental conditions of cultivation, the warm temperature is suitable for *Bulbophyllum* plants that grow with a minimum of 22°C in winter, though species from temperate regions are grown 5–10 degrees cooler [30]. Light can be moderate to bright (2000 to 3500 candle feet): a higher light, which does not damage the leaves, seems to produce a better and more frequent flowering [30]. *Bulbophyllum* species tend to prefer a minimum of repotting using fern or cork slabs, baskets, and well-draining pots and the recommended impregnation media are sphagnum moss, coconut (flakes or coconut fibres), and tree fern both horizontally and vertically, whereas the relative humidity should be around 90% inside a greenhouse [31]. High relative humidity with a high air movement rate and constant fresh air are decisive parameters for the health and successful cultivation of the plants [31]. During the growth cycle, plants need adequate nutrition using standard fertilizers at 1/4 of the concentration given for houseplants [30]. The plants are usually rapid growers, reporting no major problems with pests. Propagation is through the division of the bulbs or via seeds of excellent germination rates [31]. Manual pollination of *Bulbophyllum phalaenopsis*, *Bulbophyllum spies,* and *Bulbophyllum strontium* has so far not been successful [31].

### 3.3. Geographical Distribution

**Figure 1:**
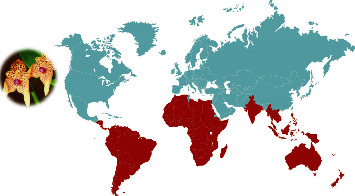
Geographical distribution of *Bulbophyllum* species.

The taxa of the genus *Bulbophyllum* are pantropical, spreading across Africa, Australia, India, Madagascar, Southeast Asia, and tropical South and Central America [32] (Figure 1).

The orchids, as epiphytes, are believed to be less adaptable to anthropogenic environmental variations than other plants [33]. The availability of the specificity host tree plays a central role in influencing the distribution and abundance of epiphytic orchids, often causing their unequal geographical distribution, even within a single tree [11].

Most of the *Bulbophyllum* species is epiphytes and is present in the virgin pantropical forest in the lower Montane forest at 1000 m above sea level [32]. However, a major number of species arise in the Indo-Malayan region [34]. Of 1000 species from India, 62 species are in the Northeastern region, 14 species in South India, and the remaining from other parts of India [35]. Madagascar is the hub of *Bulbophyllum* diversity (over 210 spp.), found mainly as epiphytes in a wide range of rainforest environments [36].

Madagascan *Bulbophyllum* molecular studies evidenced the existence of a monophyletic group of Late Miocene age with two major lineages: a species-rich core clade, mainly distributed in eastern rainforest of mid-to-high elevation (c. 800–1300 m), and a species-poor clade that is ecogeographically wide-ranging [37].

According to research, about 105 species of *Bulbophyllum* are present in China [38, 39]. *Bulbophyllum* Thouars is one of the largest genera among the orchid family, with around 2000 species in the tropical and subtropical zone of the world [40].

Up to date, Vietnam has documented 122 species of the genus *Bulbophyllum* [41]. Recent studies have allowed identifying four new species of *Bulbophyllum* in Vietnam, namely *Bulbophyllum flavescens* (sect. *Aphanobulbon*), *Bulbophyllum ovatum* (sect. *Desmosanthes*), *Bulbophyllum physocoryphum* (sect. *Macrocaulia*), and *Bulbophyllum wendlandianum* (sect. *Cirrhopetalum*) [40]. These species are endemic of the Indochinese Peninsula, except for *Bulbophyllum flavescens,* which is widely distributed in western Malesia [40]. The “vinaceous orchid” (*Bulbophyllum vinaceum* Ames and C. Schweinf) is a rare epiphytic plant endemic to the highlands of Borneo Island, such as the Crocker Range and Mt. Kinabalu of Sabah [21].

## 4. Ethnopharmacology

The usage of orchids in Ayurvedic or folklore treatment is common in many parts of the world, which raised immense attention to explore their pharmacological properties and bioactive constituents in-depth. The genus *Bulbophyllum* has an important role in many cultures acting as a religious, protective, ornamenting, cosmetic, and medicinal means. Leaves, pseudobulbs, and flowers of *Bulbophyllum* sp. have ethnobotanical importance and are used for various ailments, for both external and internal applications and administrations by tribal peoples. *B. neilgherrense* is an epiphytic plant used by the South Indian tribes to treat heart disease, leukoderma, skin allergy, and rheumatism; its pseudobulb is the most used part for traditional remedies.

The uses of *B. scaberulum* in South African traditional medicine were observed for pain-related ailments, recommending further studies to explore the chemical profile and interactions between different classes of compounds and biological/pharmacological activities.

In Cameroon, *Bulbophyllum falcatum* and *Bulbophyllum lupulinum* are used against sorcery [2]. At the same time, leaves of *Bulbophyllum falcatum* are used for predictions, and the whole plant of *Bulbophyllum simonii* is used as a luck potion [2]. *Bulbophyllumshanicum* is offered in many religious ceremonies and used by Kayaladies for ornamenting their hair [42]. *Bulbophyllum simonii* and *Bulbophyllum lilacinum* are mixed in body lotion to keep the body fresh and cool [2, 12].

Fluids from cleaned pseudobulbs of *Bulbophyllum lilacinum* are extracted by the press, kept in a sealed jar overnight, and then mixed with water and taken as a cool drink [12]. Powder of *Bulbophyllum melinostachyum* is recommended as anti-poisons [2].

In Zimbabwe, bark from species of the *Bulbophyllum* genus is tied around a fracture as a supporting pad [43]. Also, in different countries, many species of the *Bulbophyllum* genus are used as traditional herbal medicines (Table 1).

## 5. Phytochemistry

**Figure 2:**
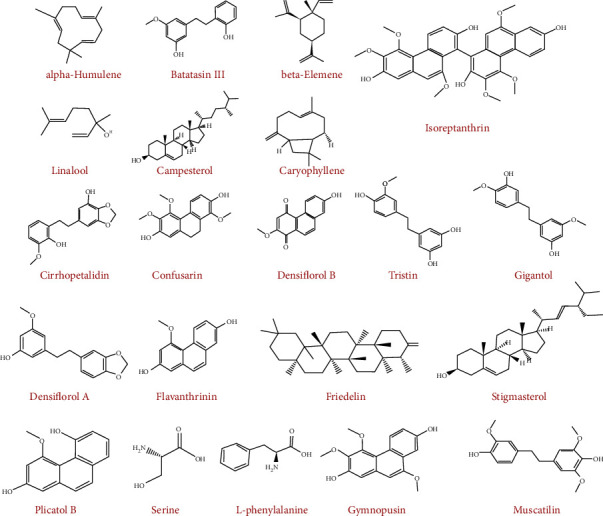
Phytochemical compounds identified in different *Bulbophyllum* species schematized by ChemDraw.

The spectroscopic analyses carried out by several investigations highlighted the phytochemicals of plants belonging to the genus *Bulbophyllum*. Although these studies are rare, their identification results have revealed a remarkable richness in the chemical composition of the genus *Bulbophyllum*. The identified compounds are shown in Table 2, and the molecular chemical structures (majority compounds) are schematized by ChemDraw and are shown in Figure 2. Note in Table 2, the 17 species belong to the genus *Bulbophyllum (B. odoratissimum, B. kaitense, B. weddellii, B. involutum, B. ipanemense, B. retusiusculum, B. neilgherrens, B. variegatum, B. vaginatum, B. protractum, B. reptans, B. cheiri, B. retusiusculum, B. kwangtungense, B. taeniophyllum, B. ambrosia, and B. echinolabium).*

Numerous chemical compounds *in B. odoratissimum* belong to different chemical families, in particular phenanthrene and phenanthraquinone. Chen et al. [56] identified 3,7-dihydroxy-2,4,6-trimethoxyphenanthrene, while the same author identified, 7-hydroxy-2,3,4-trimethoxy-9,10-dihydrophenanthrene, coelonin, densiflorol B, gigantol, batatasin III, tristin, vanillic acid, and syringaldehyde as major compounds in 2008 Moscatin [56]. Additionally, Xu et al. [59] identified bulbophythrin A and bulbophythrin B. *B. odoratissimum* was also reported to contain 5-(2-benzo[1,3]dioxole-5-ylethyl)-6-methoxy benzo[1,3]dioxole-4-ol (1) and 5-(2-benzo[1,3]dioxole-5-ylethyl)benzo[1,3]dioxole-4,7-diol [60].

The chemical compounds of *Bulbophyllum weddellii, Bulbophyllum involutum,* and *Bulbophyllum ipanemense* were characterized by Da Silva et al. [62]. In this study, several chemical compounds were identified including furfural, 2-furanomethanol, 5-methyl-2(3H)-furanone, 2,3-dihydro-4-hydroxy-2,5-dimethyl-3-furanone, 2-methoxy-phenol, maltol, 2,3-dihydro-3,5-dihydroxy-6-methyl-4H-pyrene-4-one, 1,2-benzenediol, 2,3,5,6-tetramethylphenol, 2,6-dimethoxyphenol, 4-hydroxy methyl benzoate, 4-hydroxy-3-methoxy methyl benzoate, 2,6-dimethyl-3-methoxymethyl-p-benzoquinone, tetradecanoic acid, pentadecanoic acid, and hexadecanoic acid [62].

The chemical compounds of the plant *B. kaitense* were reported by Kalaiarasan et al. [61]. The authors observed that this plant contained numerous compounds belonging to different classes such as ether compound (propane, 1,1-diethoxy), hydrocarbon (cyclopentane, 2-methylbutyl), plasticizer compound (1,2-benzenedicarboxylic acid, butyl 2-methylpropyl ester), ketone compound (2-nonanone, 9-hydroxy), aromatic alcoholic compound (2,4-dimethylcyclopentanol), alcoholic compound (3-buten-2-ol), iodo compound (nonane, 1-iodo), plasticizer compound (didodecyl phthalate), alcoholic compound (3,4-hexanediol, 2,5-dimethyl), ketone compound (fluorenone, 2,3,4,7-tetramethoxy), triterpene (squalene), bromo compound (methyl 3-bromo-1-adamantaneacetate), and aromatic compound (1,3-bis(trimethylsilyl)benzene) [77].

The identification of chemical compounds of *B. vaginatum* begun in 1997 by the study of Yuan-Wah Leong et al. [68]. This work revealed the presence of different chemical compounds belonging to several chemical family such as phenanthrenes (4,9-dimethoxyphenanthrene-2,5-diol and 4,6 dimethoxyphenanthrene-2,3,7-triol,3,4,6-trimethenanthrene-2,7-diol, 3,4-dimethoxyphenanthrene-2,7-diol (nudol), 2,4 dimethoxyphenanthrene-3,7-diol, 3,5-dimeth-oxyphenanthrene-2,7-diol, 4 methoxyphenanthrene-2,3,5-triol (fimbriol B),4-methoxyphenan-threne-2,7-diol (flavanthrinin)), less bibenzyls (3,4′-dihydroxy-5,5′-dimethoxybibenzyl and 3,3′-dihydroxy-5-methoxybibenzyl) (batatasin III), and triterpenoid friedelin [68].

In the same year, *B. protractum was found to contain numerous bioactive compounds including* bulbophyllin, bulbophyllidin, batatasin III (3,3′-dihydroxy-5-methoxy bibenzyl), 3,3′,5-trimethoxybibenzyl, aloifol-I (3′,4-dihydroxy-3,5-dimethoxybibenzyl), 3,3′-dimethoxy-4,5-methylenedioxybibenzyl, flavidin (2,7-dihydroxy-9,10-dihydro-5H-phenanthro[4,5-bcd]pyran), dihydroconiferyl alcohol, stigmasterol, and sitosterol [69]. In addition, *B. reptans* was found to be rich *in* dimeric phenanthrenes (reptanthrin and isoreptanthrin) and stilbenoids (gymnopusin, confusarin, 2,7 dihydroxy-3,4,6-trimethoxyphenanthrene, flavanthrinin, cirrhopetalanthrin) [70].

Furthermore, *B. cheiri* contains several phenylpropanoids (eugenol, methyl eugenol, cis-methyl isoeugenol, trans-methyl isoeugenol, 2-allyl-4,5-dimethoxyphenol, 5-allyl-1,2,4-trimethoxybenzene (euasarone), and trans-3,4-dimethoxycinnamyl acetate) [71], while only two phenylpropanoid (bobulretulate A, bobulretulate B) esters were identified in *B. retusiusculum* (extracts) [72]. In contrast, six dihydrodibenzoxepins (7,8-dihydro-5-hydroxy-12,13-methylenedioxy-11-methoxyldibenz[B,F]oxepin, 7,8-dihydro-4-hydroxy-12,13-methylenedioxy-11-methoxyldibenz[B,F]oxepin, 7,8-dihydro-3-hydroxy-12,13-methylenedioxy-11-methoxyldibenz[B,F]oxepin, cumulatin, densiflorol A, and plicatol B) were identified from *B. kwangtungense* [73].

The extracts of *B. retusiusculum* contain several phytochemical compounds belonging to phenylpropanoids such as retusiusine A, retusiusine B, (±)-retusiusine C, dihydroconiferyl dihydro-p-coumarate, methyl 3-(4-hydroxyphenyl) propionate, 3-(4-hydroxyphenyl)-propionic acid, dihydroferulic acid, methyl 3-(4-methoxyphenyl) propionate, 3-(3,4-dimethoxyphenyl)-2-propenal, trans-p-coumaric acid, and dihydroconiferyl alcohol [63]. In addition, bibenzyl (bulbotetusine) and flavone C-glycoside (apigenin 6-C-*α*-arabinofuranosyl 8-C-*α*-arabinopyranoside) have also been identified in this plant [64].

The phytochemical compounds of *Bulbophyllum variegatum* were also identified [66]. The finding revealed the presence of several chemical families in this plant such as alcohols (2-ethylhexanol, 2-nonanol), aldehydes (nonanal and decanal), ketones (2-heptanone, 2-nonanone), acids (acetic acid, propanoic acid), sesquiterpene hydrocarbons (beta-elemene, *(E)*-caryophyllene, alpha-humulene), nitrogenous compounds (trimethylamine, methoxyphenyloxime, indole), sulphur compounds (methyl thioacetate, benzothiazole), and aromatic compounds (toluene, *p*-cresol, *p*-cresyl acetate) [66]. *B. neilgherrens* is another medicinal species of the *genus Bulbophyllum.* Only qualitative analysis of the chemical contents of this species was carried out by Kumari et al. [65]. The results showed the presence of different chemical classes in this plant such as alkaloids, saponin glycosides, tannins, phenols, flavonoids, and steroids [65].

Both the quantitative and qualitative differences in chemical composition across species of the genus *Bulbophyllum* are due to the physiology and genetics of the species. However, other factors such as climate, soil, parts used, and phenological stages of the plants can also affect the secondary metabolite synthesis in these species.

Recent studies show that chemical composition can vary within the same species, in different environments, and in extraction systems.

## 6. Biological Activities

### 6.1. Antimicrobial and Antifungal Activities

Phytochemical screening of different solvent extracts (petroleum ether, chloroform, ethanol, and water) collected from stems of terrestrial orchid *B. kaitense* in Kolli Hills, India, confirms the presence of terpenoids, flavonoids, reducing sugars, phenols, catechins, saponins, tannins, anthraquinone, quinine, coumarin, glycosides, and carbohydrates. Ethanol and chloroform extracts show greater antifungal activity than petroleum ether and aqueous extracts, whereas the antibacterial activity in petroleum ether extract showed less effect than that reported in chloroform, ethanol, and aqueous extracts [61].

There are many orchids with different medicinal properties and antibacterial activity. *B. neilgherrense was* tested for antibacterial activity against five bacterial species (*Escherichia coli, Staphylococcus aureus, Bacillus pumilus, Pseudomonas aeruginosa*, and *Pseudomonas putida*). Ethanolic, chloroform, and aqueous extracts (concentration of 5.50 w/v) from leaves and pseudobulbs were prepared for the disk diffusion method of antimicrobial sensitivity testing. The ethanolic extract of both leaves and pseudobulbs was more effective. The bacterial species *P. aeruginosa* and *P. putida* showed greater sensitivity to the pseudobulb ethanolic extract, while the ethanolic extract from leaves was effective against *E. coli, S. aureus*, and *P. aeruginosa*. However, all extracts were less effective than standard antibiotic streptomycin, when tested with the disk diffusion method *in vitro* [78].

In another study, Fang et al. [63] identified new phenylpropanoids in tubers of *B. retusiusculum*, which were then tested for their antimicrobial activities. The antimicrobial tests were performed against *E. coli, B. subtilis*, and *Candida albicans*, where kanamycin (4 *μ*g/mL) and nystatin (4 *μ*g/mL) were positive controls for measuring antibacterial and antifungal activity potencies, respectively. Retusiusine B exhibited potent antifungal activity against *C. albicans* (16 *μ*g/mL), and (±)-retusiusine C enantiomers showed moderate antibacterial activity against *B. subtilis* (64 *μ*g/mL) [63].

*B. affine* has moderate bactericidal activity against *Staphylococcus aureus* (a common cause of skin infections) but none against *Bacillus subtilis, Klebsiella pneumonia, Escherichia coli, or Vibrio cholera* [79].

The orchid species *B. careyanum* and *B. leopardinum* are commonly used in burn treatments [80] although antimicrobial testing has not been reported for these species. In a similar study, *B. neilgherrense* exhibited moderate antibacterial activity against five infectious bacterial species [78], while promising antifungal activity against ten pathogenic fungal strains was reported from the same orchid species [81].

Phenylpropanoids isolated from *Bulbophyllum retusiusculum* exhibited moderate antibacterial activity against *Bacillus subtilis* (64 *μ*g/mL) and potent antifungal activity against *Candida albicans* (16 *μ*g/mL) [63]. *Bulbophyllum kaitense* stem extract was also effective against 10 different bacterial strains with greater sensitivity to fungal strains [61].

In the light of these results, the antibacterial activity of orchids is a good alternative for preventing/treating infections instead of using antibiotics, which have many side effects.

### 6.2. Antioxidant Activity

Natural plant products have been used as poultices and/or anti-inflammatory drugs and antioxidants for many years [82, 83].

Chinsamy et al. [84] reported antioxidant activity of *Bulbophyllum scaberulum* higher than other South African orchid species. The overall average antioxidant activity (%ANT) of *B. scaberulum* pseudobulb and root extracts was higher than 90%, which might validate the use of species to treat certain inflammatory disorders.

The antioxidant effects and inhibition of acetylcholinesterase (AchE) enzyme from several indigenous orchid species (including *B. Scaberulum)* were evaluated. In their studies, the methanolic extracts of leaves, pseudobulbs, and roots of *B. Scaberulum* (similar to other orchids the extracts) showed high antioxidant potential with 100% average antioxidant activity (ANT) as compared to the standard BHT drug (95.88%), when tested with *β*-carotene bleaching assay. They also found that the ethanol root extract at 5 and 0.5 mg/m exhibited significant mutagenic effects comparable to the 4NQO drug. Similarly, the dichloromethane extract of roots significantly inhibit AchE with the lowest IC_50_ value of 0.02 mg/ml, while the ethanol extract showed less activity against AchE.

Recently, Sun et al. [85] purified several compounds from *B. retusiusculum* whole plants. New phenanthrene, bobulretin, and two bibenzyls were evaluated against *α*-glucosidase activity *in vitro*. All three compounds showed less than 20% inhibition when tested at the final concentration of 4.37 × 10^−4^ mol/L.

Similarly, *Bulbophyllum* sp. exhibited higher antioxidant activity when compared to four other epiphytic orchids [86]. Plants of *B. kaitense* also presented good antioxidant activity and are considered a source of plant-derived antioxidants [87].

Polyphenols such as chrysin and pinobanksin found in orchid species of the genus *Bulbophyllum* have antioxidant effects [65, 88, 89], thus supporting their use in various heart diseases by the folklore traditions.

### 6.3. Anti-Inflammatory Activity

Nair et al. [54] used the pseudobulb powder of *B. neilgherrense* to examine analgesic and anti-inflammatory activities using different rat models. The pseudobulb powder mixed with honey and water revealed central analgesic activity against radiant heat-induced pain, moderate anti-inflammatory activity against carrageenan-induced acute inflammation, and mild or negligible activity against formalin-induced subacute inflammation and pain in rats. Pseudobulb contains flavonoids (chrysin and quercetin), glycosides, tannin, phenolic compounds, and calcium and may play a fundamental role in observed analgesic and anti-inflammatory activities [54].

A study run by Kumari et al. [65] reported that the pseudobulb of *B. neilgherrense* contains alkaloids, tannis, phenols, flavonoids, steroids, saponin glycosides, and reducing sugar (almost the same compounds as found earlier by Nair and his coworkers). The amounts of tannins, sugars, and alkaloids were 0.828%, 8.96%, and 0.3%, respectively (w/w). Of these, chrysin (a flavonoid) inhibited COX-2 expression and IL-6 signalling, indicating that *B. neilgherrense* pseudobulbs have anti-inflammatory potential [65].

*B. kaitense* was examined *in vitro* for its anti-inflammatory activity using the human red blood cell (HRBC) method [77]. While petroleum ether, chloroform, and aqueous extracts exhibited various anti-inflammatory activities, the ethanolic extract of *B. kaitense* pseudobulbs showed potent anti-inflammatory activity. As south Indian orchids, some South African medicinal orchids also demonstrated notable anti-inflammatory effects, suggesting their potential in treating inflammation and related disorders.

A study conducted by Chinsamy et al. [84] reported that the dichloromethane, ethanol, and aqueous root extract of *B. scaberulum* had selective and significant COX-2 inhibition effects. Inhibition was 100.00, 93.31, and 58.09%, respectively, and dichloromethane and ethanol extract IC_50_ values against COX-2 were 1.43 and 0.44 mg/ml, respectively. Surprisingly, the dichloromethane root extracts showed a greater inhibition performance than the commercial drug galantamine for COX-2. In contrast, water extracts from leaves and pseudobulbs exhibited moderate effects on COX-2, but no effect on COX-1. The COX-2 inhibition effects of *B. scaberulum* suggested that it was due to condensed tannins present in the stems and/or roots [84].

Gowlis of Karnataka use a paste of pseudobulbs of *Bulbophyllum neilgherrense Wight.* for arthritis [90]. A study validated this trait and concluded that the pseudobulb powder had central analgesic activity against radiant heat-induced pain and moderate anti-inflammatory activity against carrageenan-induced acute inflammation [54]. The authors suggest that the potential mode of action of B. neilgherrense Wight. pseudobulb was due to the presence of flavonoids (chrysin and quercetin) in the plants [65] as several flavonoids such as hesperidin, luteolin, and quercetin have anti-inflammatory and analgesic effects [91]. Similarly, chrysin and quercetin have significant analgesic and anti-inflammatory activities [92]. However, a 50% ethanolic extract of *B. gymnopus Hook f.* failed to show antimicrobial or anti-inflammatory activity and did not affect either respiration, cardiovascular system, or central nervous system, in experimental animals [93].

Aqueous pseudobulb extracts of *B. scaberulum* showed poor or no COX-1 and COX-2 inhibition [84]. Interestingly, the organic extracts (petroleum ether, dichloromethane, and ethanol) of *B. scaberulum* showed higher activity in the same study.

*Bulbophyllum kaitense* has been used in indigenous medicine by local healers of the Kolli hills. Its anti-inflammatory validation has been reported using human red blood cell (HRBC) membrane stabilization method [46]. The HRBC membrane stabilization assay uses the HRBC method as analogous to lysosomal membrane components, and thus, the inhibition of hypotonicity or heat-induced red blood cell membrane lysis may be taken as a measure of the anti-inflammatory activity mechanism of extracts.

### 6.4. Anticancer Activity

Numerous studies report that *Bulbophyllum* species have *in vitro* cytotoxicity activity. The phenanthrenes isolated from *Bulbophyllum odoratissimum* and *Bulbophyllum inconspicuum* showed significant cytotoxicity against various cancer lines such as the human leukaemia cell lines K562, HL-60, and SMMC-7721 [59, 94]. Similarly, dihydrodibenzoxepins isolated from *Bulbophyllum kwangtungense* also exhibited antitumour activities against HeLa and K562 human tumour cell lines [73]. Chen et al. [56] isolated and studied various phenolic compounds from *Bulbophyllum odoratissimum* as their inhibitory ability against the growth of human leukaemia cell lines K562 and HL-60, human lung adenocarcinoma A549, human hepatoma BEL-7402, and human stomach cancer SGC-7901. The results indicated a high activity against selective cell lines, i.e., in three compounds where densiflorol was the most active compound, followed by syringaldehyde and tristin [57]. The other compounds evaluated were weak or either inactive.

Biswas et al. [95] reported that *Bulbophyllum* sterile petroleum ether fraction induces apoptosis *in vitro* and ameliorates tumour progression *in vivo*, suggesting that the active fractions of bulbs and roots have anticancer activity likely by inducing apoptosis through the phospho-p53-dependent pathway [95].

The extract of *B. kwantungense* also exhibited antitumour activity *in vitro* against cultivated human cervical carcinoma cells (HeLa) [96]. Chen et al. [56] isolated phenanthrene derivative 3,7-dihydroxy-2,4,6-trimethoxyphenanthrene from *B. odoratissimum*, and its structure was elucidated by extensive chemical transformations and nuclear magnetic resonance (NMR) spectroscopy spectrum studies. The isolated compound showed significant cytotoxicity against the growth of human leukaemia cell lines HL-60 and K562, human stomach cancer cell line SGC-7901, human hepatoma BEL-7402, and human lung adenocarcinoma A549 with IC_50_ values of 10.02, 14.23, 1.13, 15.36, and 3.42 mg/ml, respectively.

In an investigation to evaluate the cytotoxicity of *B. kwangtungense*, Wu et al. [73] purified three new compounds, dihydrodibenzoxepins 7,8-dihydro-5-hydroxy-12,13-methylenedioxy-11-methoxydibenz[*bf*]oxepin (D5MO), 7,8-dihydro-4-hydroxy-12,13-methylenedioxy-11-methoxydibenz[*bf*]oxepin (D4MO), and 7,8-dihydro-3-hydroxy-12,13-methylenedioxy-11-methoxyldibenz[*bf*]oxepin (D3MO), along with compounds cumulatin, densiflorol A, and plicatol B. Within the compounds tested for antitumour properties, D4MO and D3MO exhibited the highest activity against HeLa cells with the IC_50_ values of 78.3 and 61.2 *μ*M, respectively, while activities against K562 tumour cells, D3MO, and densiflorol A showed the highest activity values with the IC_50_ of 64.7 and 67.6 *μ*M, respectively. In another study, two dihydrostillbenes, 3-(2-(7-methoxybenzo[d][1,3]dioxol-5-yl)ethyl)phenol (3MDP) and 6-(3-hydroxyphenethyl)benzo[d][1,3]dioxol-4-ol (6HBD), previously purified from *B. odoratissimum*, were synthesized via the Wittig–Horner reaction and used for developing nine synthetic analogues by Zhang et al. [97]. 3MDP and 6HBD together with their two analogues bearing an amino acid moiety in place of the phenolic OH of 3MDP and 6HBD were found to have significant anti-proliferative activity selective to two cancer cell lines, SGC-7901 and KB, with IC_50_ value of <10.0 *μ*M, while the other analogues showed a markedly reduced cytotoxicity towards all tumour cell lines, SGC-7901, KB, and HT-1080.

Chen et al. [57] purified nine phenolic compounds, including moscatin, 7-hydroxy-2,3,4-trimethoxy-9,10-dihydrophenanthrene, coelonin, densiflorol B, gigantol, batatasin III, tristin, vanillic acid, and syringaldehyde, from the ethyl acetate extract of *B. odoratissimum* whole plant and investigated the cell growth inhibition effects on different tumour cell lines such as human leukaemia cell lines K562 and HL-60, human lung adenocarcinoma A549, human hepatoma BEL-7402, and human stomach cancer SGC-7901. Except for three compounds such as densiflorol B, tristin, and syringaldehyde, all other compounds showed weak or no activity on tumour cells. Densiflorol B was the most active compound against all tumour cells showing the IC_50_ values ranging from 0.08 to 3.52 *μ*g/ml. Tristin displayed selective cytotoxicity against SGC-7901, with an IC_50_ value of 2.08 *μ*g/ml, whereas syringaldehyde exerted a strong activity against BEL-7402 cells with an IC_50_ value of 1.54 *μ*g/ml.

Two new dimeric phenanthrenes, bulbophythrins A and B, were then purified from *B. odoratissimum*, and their inhibitory ability against the growth of the same tumour cell lines was evaluated, as previously tested by Chen and his coworkers [57], with both compounds exerting significant cytotoxicity against all tumour cell lines [59]. However, bulbophythrin A exhibited some selective cytotoxicity against HL-60 and BEL-7402 with IC_50_ values of 1.27 × 10^−3^ and 1.22 × 10^−3^ *μ*M, respectively, whereas bulbophythrin A appeared to be most active against A549 with an IC_50_ value of 1.18 × 10^−3^ *μ*M, suggesting their potential use as a novel class of antitumour candidate in tumour disease.

Petroleum fraction, compared with the alcoholic extracts, from bulbs (PFB) and roots (PFR) of *B. sterile*, was found to be the most active in three different cancer cell lines, HCT-116, MDA-MB-231, and A549, by [95]. However, there was significant cytotoxicity in HCT-116 cells with IC_50_ values of 94.2 and 75.7 *μ*g/ml for PFB and PFR, respectively, likely attributed to the effects on the cell cycle G2/M phase with 32.6% and 49.4% arrest. In addition, PFB and PFR treatments showed 48% and 38% apoptosis, respectively, when tested with acridine orange/ethidium bromide (AO/EB) staining assay. Their apoptosis induction was carried out through phospho-p53-dependent pathway. Both fractions lowered tumour volume and increased life span and hepatic antioxidant level in Ehrlich ascites carcinoma (EAC) bearing mice, resulting in lower EAC-induced mortality.

In a different report by Yang et al. [64], two compounds, a flavone C-glycoside and bibenzyl purified from B. retusiusculum tubers, did not show evident cytotoxicity on six cancer cell lines, including HL-60, SMMC-7721, A549, MCF-7, and SW-480. Their IC_50_ value was greater than 40 *μ*M, and their noncytotoxic effects were previously thought to be attributed to their structural differences to other bibenzyl compounds previously reported to have cytotoxic effects.

### 6.5. Neuroprotective Effect

Alzheimer's disease (AD) is a progressive neurodegenerative disorder associated with memory impairment and cognitive deficit [98]. Various mechanisms, such as AChE inhibition, modification of monoamine levels, anti-amyloid aggregation, and antioxidant activities, are the strategies that have been employed for the amelioration of AD symptoms [99, 100]. Of these, one of the major approaches has involved addressing the levels of acetylcholine in the AD-depressed brain using AChE inhibitors [101]. Most of the currently available drugs are AchEi, and some are related to natural products with an important therapeutic strategy for the treatment of AD. Many research groups have focused their studies on naturally occurring compounds from plants as potential sources of either new or more effective AChEi. Due to the presence of flavonoids and tannins, orchid extracts that display significant effects in anti-inflammatory, antioxidant, and AChE inhibitory assays could be potential natural plant products in the inflammatory treatment of neurodegenerative disorders.

Chinsamy et al. [84] reported anticholinesterase activity (EC_50_) for different extraction solvents as 0.02 ± 0.00 and 0.26 ± 0.007 mg/ml, respectively, in dichloromethane and ethanol root extracts of *B. scaberulum*. The authors also found that *B. scaberulum* root extract effectively inhibited AChE as compared to the commercial product galantamine.

### 6.6. Other Activities

Myanmar women prepare a hair tonic and shampoo by mixing ground pseudobulbs of various species of *Bulbophyllum* with pulverized bark, seeds, and fruit (species not identified) when washing their hair. This mixture is claimed to cure dandruff, promote hair growth, and improve hair colour [102].

Imbricatin, a stilbenoid isolated previously from *Bulbophyllum* and other orchid genera, was one of three isolated stilbenoids recommended for use as skin photoprotectants based on their antioxidant, anti-inflammatory, and immunomodulatory effects [103].

**Figure 3:**
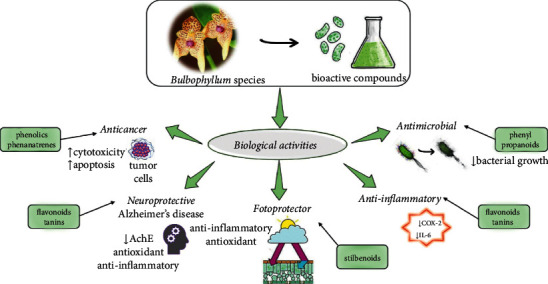
Illustrative scheme with the most important biological activities of *Bulbophyllum* species and correlations with their bioactive compounds. Abbreviations and symbols: ↑, increase, ↓, decrease, COX-2, cyclooxygenase 2, IL-6, interleukin-6, and AchE, acetylcholinesterase.

A summarized scheme of the most representative biological activities is summarized in Figure 3.

## 7. Safety Data

No reports were found in the literature concerning health safety issues after the consumption of *Bulbophyllum.* This plant has been used in ethnomedicine and folk medicine by villagers in different conditions for ages; however, the toxicity profile of the *Bulbophyllum* species is still undiscovered. With the increasing cases of poisoning associated with the use of herbal medicines in many parts of the world in recent times [104], it is essential to ensure safety through toxicity assessment alongside active pharmacovigilance to promote their safe use and protect public health. Therefore, it is necessary to identify the risks associated with the use of such herbal plants, and in this regard, the safety of these products has become an issue of great public health importance and thus a key moment for considering it in pharmacovigilance systems.

## 8. Limitations and Future Perspective

The data from this review validate the use of certain orchid species in ethnopharmacology for various conditions. More comprehensive studies of the bioactive compounds of several *Bulbophyllum* species would open a new perspective on the relationships between chemical profiles, interactions between different classes of chemical compounds, biological properties, and correlation with geographical location. Therapeutic limitations of *Bulbophyllum* species are due to ignorance of long-term adverse effects, toxicity, and mutagenic potential. Future experimental pharmacological research is needed to further investigate the molecular mechanisms and action targets of the bioactive compounds contained in *Bulbophyllum* species. Another limitation is the lack of clinical trials.

## 9. Conclusion

In recent years, the genus *Bulbophyllum* is gaining the attention of plant researchers because of its rich phytochemical profile and variety of biological activities reported across species of the genus. Most *Bulbophyllum* species are epiphytic and found in habitats that range from subtropical dry forests to wet montane cloud forests. Plants belonging to the genus *Bulbophyllum* are mostly epilithic herbs and sympodial with roots creeping over the surface of the substrate or aerial, filamentous to fibrous. The genus *Bulbophyllum* has been utilized for protective, religious, cosmetic, ornamenting, and medicinal applications. Numerous investigations report the phytochemical compounds extracted from *Bulbophyllum* root, pseudobulb, and leaf, and their biological effect in traditional medicine treatments. Several phytochemical compounds of biological interest such as flavonoids, sterols-terpenoids, and phenolic acids have been reported in *Bulbophyllum* species. As summarized in the present review, the phytochemical composition of species of the genus *Bulbophyllum* offers insights into providing information on the antioxidant, anti-inflammatory, cytotoxic, antimicrobial, anticancer, and anticholinesterase activity. Furthermore, investigations are warranted to extract the various health-promoting phytochemicals and to identify their bioactivities, which will help to boost the utilization of *Bulbophyllum* species.

## Figures and Tables

**Table 1:** Traditional and folk medical usage of the *Bulbophyllum* species.

*Bulbophyllum* species	Country	Usage	References
*Bulbophyllum albidum*	India	Strengthening of a weak uterus for conception	[44]

*Bulbophyllum barbigerum*	Cameroon	Side pain (whole plant), ear pain (leaves)	[2]

*Bulbophyllum calyptratum*	Cameroon	Skin diseases (measles, poxes abscesses, rashes) (leaves), wounds, burns (whole plant)	[2]

*Bulbophyllum careyanum*	Nepal	Burns (pseudobulb), abortion, and recovery during childbirth (leaves)	[3]
	India	Burns (pseudobulb), abortion, and recovery during childbirth (leaves)	[45]

*Bulbophyllum cariniflorum*	Not specified	Induce abortion (root)	[10]

*Bulbophyllum intertextum*	Cameroon	Side pain (whole plant)	[2]

*Bulbophyllum kaitesse*	India	Cancer, inflammatory, bacterial infection (pseudobulb)	[46]

*Bulbophyllum kwangtungense*	China	Pulmonary tuberculosis, bleeding, fever (tuber)	[47]
	Japan	Pulmonary tuberculosis, bleeding, fever (tuber)	[47]

*Bulbophyllum leopardinum*	Nepal	Burns	[48]
	India	Burns	[45]

*Bulbophyllum lilacinum*	Bangladesh	Tiredness, anxiety, aphrodisiac, inflammation, rheumatism, hypertension, diabetes, anemia, tuberculosis, cough, asthma, jaundice, heavy menstruation, leucorrhoea, eye disease, wound (pseudobulb, whole plant)	[10, 49, 50]

*Bulbophyllum modestum*	Thailand	Ear infection (stem)	[51]

*Bulbophyllum mutabile*	Malaysia	Fever (leaves)	[52]

*Bulbophyllum neilgherrense*	India	Heart diseases, rheumatism, leukoderma (pseudobulb), weakness (juice), tuberculosis, chronic inflammation, fractures, scabies (whole plant)	[53–55]
	Bangladesh	Tonic	[50]

*Bulbophyllum odoratissimum*	Bhutan	Tuberculosis, chronic inflammation, fracture (whole plant)	[47]
	Burma	Tuberculosis, chronic inflammation, fracture (whole plant)	[47]
	China	Cough, toothache, tuberculosis, chronic inflammation, fracture (whole plant)	[47], 15]
	India	Tuberculosis, chronic inflammation, fracture (whole plant)	[45, 47]
	Laos	Tuberculosis, chronic inflammation, fracture (whole plant)	[47]
	Nepal	Tuberculosis, chronic inflammation, fracture (whole plant)	[47, 48]
	Thailand	Tuberculosis, chronic inflammation, fracture (whole plant)	[47]
	Vietnam	Tuberculosis, chronic inflammation, fracture (whole plant)	[47]

*Bulbophyllum pumilum*	Cameroon	Epilepsy (whole plant)	[2]

*Bulbophyllum sterile*	India	Rheumatism, swellings (pseudobulb)	[45]

**Table 2:** Phytochemistry of *Bulbophyllum* species.

*Bulbophyllum* species	Chemical classes	Compounds	References
*Bulbophyllum Odoratissimum*	Phenanthrene	3,7-Dihydroxy-2,4,6-trimethoxyphenanthrene	[56]
		Moscatin, 7-hydroxy-2,3,4-trimethoxy-9,10-dihydrophenanthrene, coelonin, densiflorol B, gigantol, batatasin III, tristin, vanillic acid, syringaldehyde	[57]
	Phenanthraquinone	Bulbophyllanthrone	[58]
	Biphenanthrenes	Bulbophythrin A and bulbophythrin B	[59]
	Dihydrostilbenes	5-(2-Benzo[1,3]dioxole-5-ylethyl)-6-methoxy benzo[1,3]dioxole-4-ol (1) and 5-(2-benzo[1,3]dioxole-5-ylethyl)benzo[1,3]dioxole-4,7-diol	[60]

*Bulbophyllum kaitense*	Ether compound	Propane, 1,1-diethoxy	[61]
	Hydrocarbon	Cyclopentane, (2-methylbutyl)	
	Plasticizer compound	1,2-Benzenedicarboxylic acid, butyl 2-methylpropyl ester	
	Ketone compound	2-Nonanone, 9-hydroxy	
	Aromatic alcoholic compound	2,4-Dimethylcyclopentanol	
	Alcoholic compound	3-Buten-2-ol	
	Iodo compound	Nonane, 1-iodo	
	Plasticizer compound	Didodecyl phthalate	
	Alcoholic compound	3,4-Hexanediol, 2,5-dimethyl	
	Ketone compound	Fluorenone, 2,3,4,7-tetramethoxy	
	Triterpene	Squalene	
	Bromo compound	Methyl 3-bromo-1-adamantaneacetate	
	Aromatic compound	1,3-bis(Trimethylsilyl)benzene	

*Bulbophyllum weddellii*		Furfural, 2-furanomethanol, 5-methyl-2(3H)-furanone, 2,3-dihydro-4-hydroxy-2,5-dimethyl-3-furanone, 2-methoxy-phenol, maltol, 2,3-dihydro-3,5-dihydroxy-6-methyl-4H-pyrene-4-one, 1,2-benzenediol, 2,3,5,6-tetramethylphenol, 2,6-dimethoxyphenol, 4-hydroxy methyl benzoate, 4-hydroxy-3-methoxy methyl benzoate, 2,6-dimethyl-3-methoxymethyl-p-benzoquinone, tetradecanoic acid, pentadecanoic acid, hexadecanoic acid	[62]

*Bulbophyllum involutum*		Furfural, 2-furanomethanol, 5-methyl-furfural, 2,3-dihydro-4-hydroxy-2,5-dimethyl-3-furanone, 2-methoxy-phenol, 2,3-dihydro-3,5-dihydroxy-6-methyl-4H-pyrene-4-one, 1,2-benzenediol, 5-hydroxymethyl-2-furan-carboxyaldehyde, tetradecanoic acid, pentadecanoic acid, 14-methyl methyl pentadecanoate, 9-hexadecenoic acid, hexadecanoic acid	[62]

*Bulbophyllum ipanemense*		Hexanal, 2-furanomethanol, 2-pentyl-furan, 1,2-benzenediol, 2,4-decadienal-(E,Z), 2,3,5,6-tetramethylphenol, 4-decadienal-(E,E), 2,6-dimethoxyphenol, tridecanone, tridecanol, methyl tetradecanoate, tetradecanoic acid, pentadecanoic acid, hexadecanoic acid, ethyl hexadecanoate	[62]

*Bulbophyllum retusiusculum*	Phenylpropanoids	Retusiusine A, retusiusine B, (±)-retusiusine C, dihydroconiferyl dihydro-p-coumarate, methyl 3-(4-hydroxyphenyl) propionate, 3-(4-hydroxyphenyl)-propanoic acid, dihydroferulic acid, methyl 3-(4-methoxyphenyl) propionate, 3-(3,4-dimethoxyphenyl)-2-propenal, trans-p-coumaric acid, dihydroconiferyl alcohol	[63]
	Bibenzyl	Bulbotetusine	[64]
	Flavone C-glycoside	Apigenin 6-C-*α*-arabinofuranosyl 8-C-*α*-arabinopyranoside	

*Bulbophyllum neilgherrens*	Alkaloids	+	[65]
	Saponin glycosides	+	
	Tannins	+	
	Phenols	+	
	Flavonoids	+	
	Steroids	+	
	Reducing sugar	+	

*Bulbophyllum variegatum*	Alcohols	2-Ethylhexanol, 2-nonanol	[66]
	Aldehydes	Nonanal, decanal	
	Ketones	2-Heptanone, 2-nonanone	
	Acids	Acetic acid, propanoic acid	
	Sesquiterpene hydrocarbons	Beta-elemene, (E)-caryophyllene, alpha-humulene	
	Nitrogenous compounds	Trimethylamine, methoxyphenyloxime, indole	
	Sulphur compounds	Methyl thioacetate, benzothiazole	
	Aromatic compounds	Toluene, p-cresol, p-cresyl acetate	

*Bulbophyllum vaginatum*		Biphenanthrene, phenanthro[4,3-b]furan derivative	[67]
	Phenanthrenes	4,9-Dimethoxyphenanthrene-2,5-diol and 4,6 dimethoxyphenanthrene-2,3,7-triol, 3,4,6-trimethenanthrene-2,7-diol, 3,4-dimethoxyphenanthrene-2,7-diol (nudol), 2,4-dimethoxyphenanthrene-3,7-diol, 3,5-dimeth-oxyphenanthrene-2,7-diol, 4-methoxyphenanthrene-2,3,5-triol (fimbriol B), 4-methoxyphenan-threne-2,7-diol (flavanthrinin)	[68]
	Dihydrophenanthrenes	4-Methoxy-9,10-dihydrophenanthrene-2,3,7-triol and 4,6-dimethoxy-9,10-dihydrophenanthrene-2,3,7-trio, 9,10-dihydrophenanthrenes 3,4,6-trimethoxy-9,10-dihydrophenanthrene-2,7-diol, 4-methoxy-9,10-dihydrophenanthrene-2,7-diol (coelonin), 3,5-di-methoxy-9,10-dihydrophenanthrene-2,7-diol (6-methoxycoelonin), 3,4-dimethoxy-9,10-dihydrophenanthrene-2,7-diol (erianthridin)	
	Triterpenoid	Friedelin	
	Bibenzyls	3,4′-Dihydroxy-5,5′-dimethoxybibenzyl and 3,3′-dihydroxy-5-methoxybibenzyl (batatasin III)	

*Bulbophyllum protractum*		Bulbophyllin, bulbophyllidin, batatasin III (3,3′-dihydroxy-5-methoxy bibenzyl), 3,3′,5-trimethoxybibenzyl, aloifol-I (3′,4-dihydroxy-3,5-dimethoxybibenzyl), 3,3′-dimethoxy-4,5-methylenedioxybibenzyl, flavidin (2,7-dihydroxy-9,10-dihydro-5H-phenanthro[4,5-bcd]pyran), dihydroconiferyl alcohol, stigmasterol, and sitosterol	[69]

*Bulbophyllum reptans*	Dimeric phenanthrenes	Reptanthrin and isoreptanthrin	[70]
	Stilbenoids	Gymnopusin, confusarin, 2,7 dihydroxy-3,4,6-trimethoxyphenanthrene, flavanthrinin, cirrhopetalanthrin	

*Bulbophyllum cheiri*	Phenylpropanoids	Eugenol, methyl eugenol, cis-methyl isoeugenol, trans-methyl isoeugenol, 2-allyl-4,5-dimethoxyphenol, 5-allyl-1,2,4-trimethoxybenzene (euasarone), trans-3,4-dimethoxycinnamyl acetate	[71]

*Bulbophyllum retusiusculum*	Phenylpropanoid esters	Bobulretulate A, bobulretulate B	[72]

*Bulbophyllum kwangtungense*	Dihydrodibenzoxepins	7,8-Dihydro-5-hydroxy-12,13-methylenedioxy-11-methoxyldibenz[ B,F]oxepin, 7,8-dihydro-4-hydroxy-12,13-methylenedioxy-11-methoxyldibenz[ B,F]oxepin, 7,8-dihydro-3-hydroxy-12,13-methylenedioxy-11-methoxyldibenz[ B,F]oxepin, cumulatin, densiflorol A, and plicatol B	[73]

*Bulbophyllum taeniophyllum*	Bibenzyls	Batatasin III and cirrhopetalidin	[74]

*Bulbophyllum ambrosia*		Moscatin,3,7-dihydroxy-2,4-dimethoxyphenanthrene, 2,7-dihydroxy-3,4-dimethoxy-phenanthrene, 2,5-dihydroxy-4-methoxy-9,10-dihydrophenanthrene,ephemeranthol-A, 3,7-dihydroxy-2,4-dimethoxy-9,10-dihydrophenanthrene, 2,4-dihydroxy-7-methoxy-9,10-dihydrophenanthrene, coelonin, rotundatin,4′,5-dihydroxyl-3,3′-dimethoxybibenzyl, moscatilin, batatasin III, and tristin	[75]

*Bulbophyllum echinolabium*	Diptera attractants	Cholest-5-en-3-ol, glycerol 1-palmitate, hexadecane, tridecane, decanal, nonanal, undecane, beta-linalool, limonene, 2-hexenal	[76]
	Amino acids	L-Phenylalanine, serine, norleucine, L-threonine	
	Saccharides	Alpha-D-glucopyranoside, D-turanose, sucrose, D-glucose, hydroquinone-beta-d-glucopyranoside, D-galactose, glucopyranose, 2,3,4-trihydroxybutyric acid, glycoside, alpha-methyl, ribonic acid, D-xylopyranose, D-(−)-tagatofuranose, inositol, D-ribofuranose, erythritol, D-(+)-xylose, myo-inositol, fructose	
	Lipids	Stigmasterol, campesterol, cholesterol, 1-monopalmitin	
	Others	Cyanuric acid, malic acid, 1-cyclohexene-1-carboxylic acid, 3,4,5-hydroxy, citric acid, alpha-hydroxyglutaric acid, mannonic acid, 1,4-lactone, benzoic acid, 3-methoxy, beta-hydroxy-beta-methylglutaric acid, p-hydroxybenzoic acid, pantothenic acid, alpha-aminoadipic acid	

## Data Availability

The data supporting this review are from previously reported studies and datasets, which have been cited. The processed data are available from the corresponding author upon request.
